# Major medical causes by breed and life stage for dogs presented at veterinary clinics in the Republic of Korea: a survey of electronic medical records

**DOI:** 10.7717/peerj.5161

**Published:** 2018-07-03

**Authors:** Eunju Kim, Changyong Choe, Jae Gyu Yoo, Sang-Ik Oh, Younghun Jung, Ara Cho, Suhee Kim, Yoon Jung Do

**Affiliations:** Division of Animal Diseases & Health, National Institute of Animal Science, Rural Development Administration, Wanju-gun, Jeollabuk-do, Republic of Korea

**Keywords:** Major medical cause, Dogs, Age, Breed, Electronic medical record

## Abstract

**Background:**

Age and breed are considered the greatest risk factors for disease prevalence and mortality in companion dogs. Understanding the prevalence of diseases, in relation to age and breed, would support appropriate guidance for future health care strategies and provide useful information for the early diagnosis of diseases. The purpose of this study was to investigate the major medical causes for dogs visiting primary-care veterinary clinics in the Republic of Korea, stratified by age and breed.

**Methods:**

A total of 15,531 medical records of canine patients were analyzed from 11 veterinary clinics who shared data from January 1, 2016 to December 31, 2016. An electronic medical record (EMR) system was used for data collection, which included the animal identification number, age, breed, gender, neuter status, clinical information, and diagnosis. EMR data were classified using the International Classification of Disease system from the World Health Organization; presenting signs or diagnoses were identified according to breed and life stage.

**Results:**

Within the age groups, preventive medicine (16.7% confidence intervals (CI) [15.9–17.5]) was the most common cause for clinic visits for the <1 year and 1–3 year groups. Additionally, neutering surgery (6.6% CI [6.0–7.1]) and patella luxation (1.4% CI [1.8–2.7]) were frequently performed in these age groups. In the 4–6 year group, otitis externa (8.8% CI [7.8–10.0]) and dermatitis or eczema (8.5% CI [7.5–9.6]) were common medical problems. In older dogs (>10 year), the prevalences of heart disease, kidney disease, Cushing’s disease, and mammary tumors were higher than in the other age groups. Small and toy breed dogs comprised 67.7% of all dogs in this analysis. For all breeds, otitis externa, dermatitis or eczema, vomiting, and diarrhea were common medical problems.

**Discussion:**

This study identified the most common medical disorders and differences in prevalences of diseases, according to age and breeds. The information from EMRs for dogs visiting primary-care veterinary clinics can provide background knowledge that is required to enable a better understanding of disease patterns and occurrence by age and breeds. The information from this study could enable the creation of strategies for preventing diseases and enable the identification of health problems for more effective disease management in companion dogs.

## Introduction

Worldwide, companion animals have become an important part of the modern family. Owners who consider companion animals to be family members, expect their dogs to have good health. Changes in living environments, improvements in nutrition, and the development of veterinary medical technology, including the prevention of infectious diseases, as well as better diagnostic approaches and disease management, have resulted in a gradual increase of the average lifespan of the companion dog (Butterwick, 2015; Guglielmini, 2003; Sabnis & Rathbone, 2013). However, as the dog lifespan has increased, chronic and degenerative diseases have become more prevalent (Bonnett et al., 2005; Bonnett & Egenvall, 2010; Fleming, Creevy & Promislow, 2011).

Previous survey studies focused on investigating the prevalence of specific diseases, such as human-animal interaction diseases, infectious diseases, or individual disease processes, such as neoplasms (Balcha & Abdela, 2017; Miller et al., 2016). In recent years, various investigators have used demographic information of companion dogs to assess health issues and disease prevalences within particular populations. In addition, mortality and morbidity in a variety of countries have been reported, stratified by age and breed (Bonnett & Egenvall, 2010; Egenvall et al., 2005; Fleming, Creevy & Promislow, 2011; Proschowsky, Rugbjerg & Ersbøll, 2003). Large dog breeds have been noted to exhibit a shorter expected lifespan than small dog breeds (Fleming, Creevy & Promislow, 2011; Michell, 1999; Proschowsky, Rugbjerg & Ersbøll, 2003); further, mixed breed dogs have a higher average lifespan than purebred dogs, except for Poodles and Border Collies (O’Neill et al., 2013; Proschowsky, Rugbjerg & Ersbøll, 2003). A previous report found that male dogs tended to have higher mortality rates due to trauma, whereas female dogs showed higher rates of neoplasms (Egenvall et al., 2005). Some breeds, such as Golden Retrievers and Boxers, die of cancer more frequently than other breeds (Fleming, Creevy & Promislow, 2011). These reports show that breed and age are major risk factors for mortality in dogs.

Although the AAHA–AVMA (The American Animal Hospital Association and the American Veterinary Medical Association) Canine Preventive Healthcare Guideline recommends at least one annual health checkup, it is not uncommon for pet owners in Korea to visit a veterinary clinic for health checkup only in the context of signs of illness, due to the owner’s busy daily life; some owners may not notice illness-related changes in behavior in their pets. Moreover, some types of diseases, such as heart disease, often show no signs, even when the disease has progressed and the patient has been significantly affected (Atkins et al., 2009). For these reasons, dog owners and veterinarians require guidelines to improve early diagnoses and prevent diseases throughout all life stages of the dog. Understanding the prevalence of diseases, according to breed and age-related disease patterns, would support better guidance for future health care strategies and provide useful information for more effective early diagnoses.

The purpose of this study was to investigate the causes of major medical conditions, in relation to the breeds and life stages, of dogs visiting primary-care veterinary clinics in the Republic of Korea (ROK), and to provide basic data for the establishment of more effective health programs for dogs. This study describes the demographics of companion dogs based on veterinary medical records at primary-care clinics.

## Materials and Methods

A total of 44,782 medical records of canine patients were analyzed from 11 selected primary-care veterinary clinics, for the period from January 1, 2016 to December 31, 2016.

### Data collection

Participating primary veterinary clinics in this study were randomly selected after an interview with the practitioner because of their even distribution by individual districts: the five primary veterinary clinics are in Eunpyeong-gu, Gangseo-gu, Guro-gu, Gwanak-gu, and Mapo-gu in Seoul (capital city in ROK) and the six primary veterinary clinics are in Wanshan-gu and Deokjin-gu in Jeonju (metropolitan city located middle-western in ROK). We included no specialists, referral clinics, or teaching hospitals in this study. Before collecting data for this survey, data use agreements were signed by veterinary practitioners from each clinic when they agreed to provide their medical records. The data included clinical information, such as presenting signs or diagnoses of major health problems, reasons for the visit, and related clinical procedures. All clinical data were recorded by the veterinarians at their practices. A proprietary electronic medical record system (EMR; Into Vet., Inc, Into CNS, Seoul, ROK) was used for data collection; it included the animal identification number, age, breed, gender, neuter status, clinical information (clinical signs, client complaints, lab procedures), and diagnosis. The data collection procedure was performed in cooperation with the EMR software company. The company extracted the data from the veterinary medical records, excluding any personal information, into an Excel file (Microsoft Office Excel 2010; Microsoft Corp., Redmond, WA, USA) for use in this study.

### Data nomenclature and classification

Among the collected data, services not related to medical care, such as grooming and boarding services, were excluded from the analysis. Repeated ongoing events within individual cases were excluded to avoid duplication. However, if a patient had two different diseases, those were recorded as two diseases. The classification includes two general categories of International Classification of Disease (ICD) for human disease. Clinical notes and diagnosis terms included in the data were reviewed in detail by a veterinary researcher with clinical experience and were reworked as a list of terms using the most appropriate the standard nomenclature of veterinary disease and operations. The most definitive diagnostic term recorded for each disorder within an individual patient’s record was manually classified using the ICD from the World Health Organization (ICD-10 version, WHO). For example, otitis externa was classified as “Diseases of the ear or mastoid process,” and heartworm was classified as “Certain infectious or parasitic diseases.” Consequences involving external causes, such as trauma including injury, accident involving fracture, and wound including lacerations, tear and bit wounds that involved by soft tissues were classified within “Injury, poisoning and certain other consequences of external causes.” Some of the terms combined nomenclature which is broad informed terms were used in this study. For example, the term “diarrhea” included watery diarrhea, mucosae diarrhea, bloody diarrhea, and melena. The term “vomiting” included vomiting, regurgitation, and hematemesis. Most records were as classified in a main category of ICD and were not grouped into sub-categories. Dental problems, such as gingivitis and periodontal disease, were grouped within “Dental disorder.” Vaccinations for infectious diseases and heartworm prevention were grouped within “Preventive medicine,” because of this category’s importance in veterinary practice. Additionally, male castration and female ovariohysterectomy were grouped within “Neutering surgery” when there was no underlying disease. Causes of death or euthanasia were not recorded.

### Age and breed classification

To investigate the prevalence of major medical conditions, the dogs were classified into groups, according to their breeds. Age was divided into seven groups; <1 year, 1–3 years, 4–6 years, 7–9 years, 10–12 years, 13–15 years, >16 years of age. Ages that were unknown were analyzed for age profile, but were not included in the average age calculation and prevention of disease for individual age groups. The top 20 most common disorders were analyzed, according to age groups, to investigate the major medical causes for visiting the veterinary clinic.

Because there were too many breeds to appropriately describe all of them (we observed over 80 individual breeds in the collected data), we excluded breeds with <500 cases of complete data. As a result, only six dog breeds (Maltese, Poodle, Pomeranian, Shih Tzu, Yorkshire Terrier, Chihuahua) and mixed breeds were used in the final analysis. Each of the presenting signs or diagnoses was analyzed, stratified by breeds, to investigate the prevalence of diseases.

### Statistics

Demographic variables were statistically described for the overall study population, according to age and breeds, and were visualized using GraphPad Prism version 5.01 (GraphPad Software, San Diego, CA, USA). The percentages of major medical reasons for clinic visits with 95% confidence intervals (CI) were calculated for twenty major medical conditions, according to age and breeds. Prevalence values for age and breeds were compared statistically, using SPSS software version 21 (IBM, Armonk, NY, USA). For all analyses, *p* < 0.05 was the threshold for statistical significance using Pearson’s chi-square test.

## Results

A total of 44,782 EMRs of dog patients were analyzed from veterinary clinics who shared medical data for the period from January 1, 2016 to December 31, 2016. When records for non-medical services and repeat visits (29,251, 65.3%) were excluded, a total of 15,531 EMRs and 11,085 individual dogs were analyzed. The proportion of dogs with more than one disease was 4,446, 28.6%.

### Age and breed distribution

**Figure 1:**
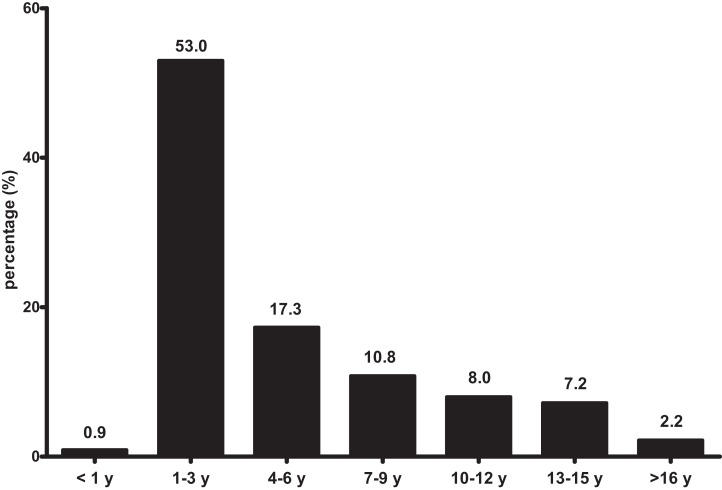
Age profile of dogs to attend small animal clinics. Study site: y, years.

The age profile of the dogs is presented in Fig. 1. The median age of the dogs was 4.8 years during the study period (Minimum value—maximum value: 0.9–20.6 years). The most frequent age group for visiting clinics was 1–3 years (53.0%). The percentage of dogs >10 years of age was 17.3%.

**Figure 2:**
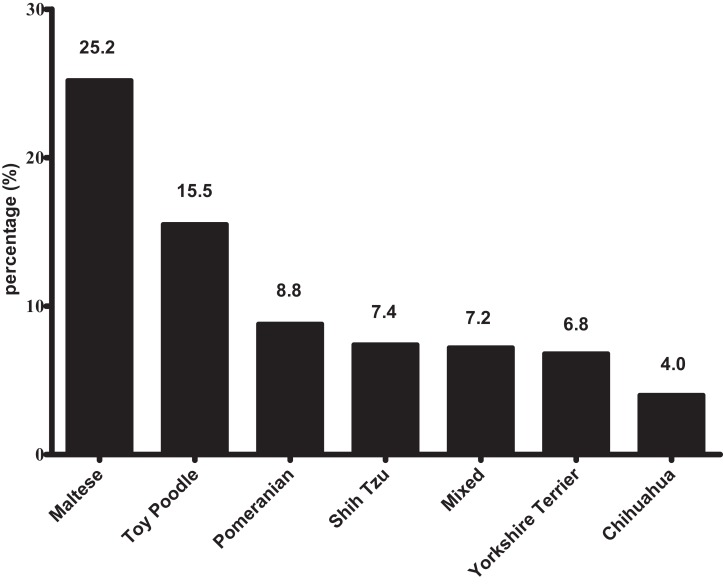
The most popular breeds (including mixed breeds) visiting small animal clinics.

The most common breeds (including mixed breeds) visiting the clinics included Maltese (25.2%), Toy Poodle (15.5%), Pomeranian (8.8%), Shih Tzu (7.4%), Yorkshire Terrier (6.8%), Chihuahua (4.0%), and mixed breed (7.2%) (Fig. 2). These six small/toy breeds comprised 67.7% of the total dogs investigated.

### Classification by ICD category

**Figure 3:**
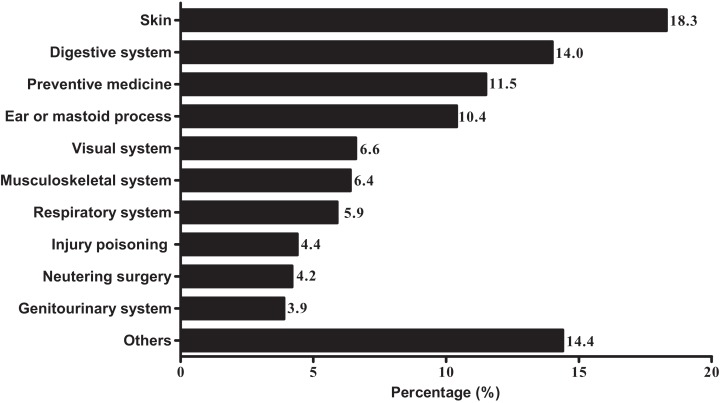
Prevalence of the most common disorders of dogs presented at veterinary clinics. Classification using the International Classification of Diseases (ICD) from the World Health Organization.

Using the ICD categories, we established 27 different disorder categories. We included a category “Others” for data where the number of cases was less than 500. Figure 3 shows the 10 most common disorders, which included: Diseases of the skin (18.3%); Diseases of the digestive system (14.0%); Preventive medicine (11.5%); Diseases of the ear or mastoid process (10.4%); Diseases of the visual system (6.6%); Diseases of the musculoskeletal system or connective tissue (6.4%); Diseases of the respiratory system (5.9%); Injury, poisoning or certain other consequences of external causes (4.4%); Neutering surgery (4.2%); Diseases of the genitourinary system (3.9%); and Others (14.4%).

### Classification by presenting signs or diagnoses

**Figure 4:**
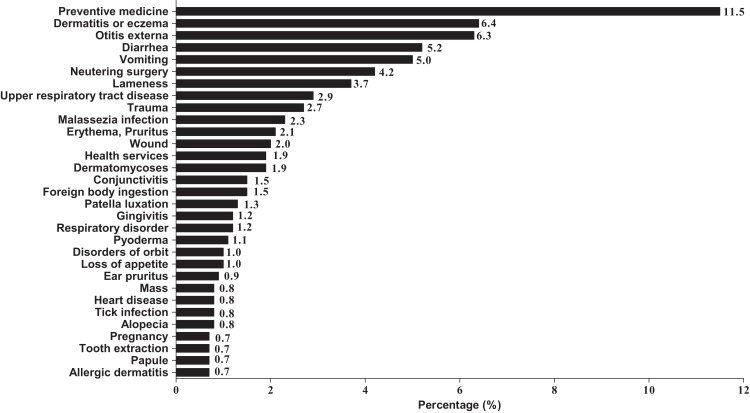
The top 31 most common causes of dogs to attend small animal clinics. Classification by presenting signs or diagnoses.

We selected the top 31 most common causes for visiting the clinics and removed classified presenting signs or diagnoses that comprised <100 data points. The most common cause was preventive medicine (11.5%), followed by dermatitis or eczema of the skin (6.4%), otitis externa (6.3%), diarrhea (5.2%), vomiting (5.0%), and neutering surgery (4.2%). These data, including the percentages of other causes, are presented in Fig. 4.

### Major medical causes for visiting veterinary clinic by age groups

Table 1 shows the percentage and CI of common disorders and other medical causes according to age. Briefly, for <1-year-old dogs, these were preventive medicine 39.0% (95% CI [31.1–47.3]), diarrhea 11.8% (95% CI [7.3–18.2]), and vomiting 5.1% (95% CI [2.2–10.7]). Additionally, infectious diseases, such as those caused by endoparasites 2.9% (95% CI [0.9–7.8]), canine parvovirus 1.5% (95% CI [0.2–5.7]), and coronavirus 1.5% (95% CI [0.2–5.7]) occurred more commonly in dogs <1-year old. For 1-to-3-year-old dogs, preventive medicine 16.7% (95% CI [15.9–17.5]) was most common cause for visiting the clinics. Neutering surgery 6.6% (95% CI [6.0–7.1]) was one of the most frequently performed procedures. For 4-to-6-year-old dogs, otitis externa 8.8% (95% CI [7.8–10.0]) was the most common medical problem, followed by dermatitis or eczema 8.5% (95% CI [7.5–9.6]) and preventive medicine 7.7% (95% CI [6.7–9.6]). Additionally, gingivitis 2% (95% CI [1.5–2.6]) was observed in the rank table for this age group. For dogs >7 years old, the most common medical problem was dermatitis or eczema, followed by otitis externa. In older dogs (≥10 years old), some diseases, such as heart disease, kidney disease, Cushing’s disease, and mammary tumors occurred more often than in other age groups.

**Table 1:** The top 20 most common medical reason in canine patients visiting primary-care veterinary clinics.

	Percentage of each medical condition in each age group (95% confidence interval)						
Rank	<1 year	1–3 years	4–6 years	7–9 years	10–12 years	13–15 years	>16 year
1	Preventive medicine 39 (31.1–47.3)	Preventive medicine 16.7 (15.9–17.5)	Otitis externa 8.8 (7.8–10.0)	Dermatitis or eczema 9.2 (7.9–10.7)	Dermatitis or eczema 7.4 (5.9–8.9)	Dermatitis or eczema 6.2 (4.8–7.8)	Dermatitis or eczema 5.6 (3.5–8.7)
2	Diarrhea 11.8 (7.3–18.2)	Neutering surgery 6.6 (6.0–7.1)	Dermatitis or eczema 8.5 (7.5–9.6)	Otitis externa 8.2 (6.9–9.6)	Otitis externa 5.6 (4.3–7.0)	Otitis externa 5.3 (4.0–6.8)	Otitis externa 3.8 (2.1–6.6)
3	Vomiting 5.1 (2.2–10.7)	Diarrhea 6.3 (5.7–6.8)	Preventive medicine 7.7 (6.7–8.8)	Lameness 4.5 (3.6–5.6)	Lameness 4.5 (3.4–5.8)	Heart disease 5.3 (4.0–6.8)	Vomiting 3.8 (2.1–6.6)
4	Health services 4.4 (1.8–9.7)	Vomiting 5.9 (5.3–6.4)	Diarrhea 4.6 (3.8–5.4)	Diarrhea 4.5 (3.5–5.6)	Vomiting 3.9 (2.9–5.1)	Lameness 3.5 (2.5–4.7)	URTD 3.8 (2.1–6.6)
5	URTD 3.7 (1.3–8.8)	Otitis externa 5.5 (5.0–6.0)	Vomiting 4.5 (3.7–5.3)	Preventive medicine 4.1 (3.2–5.1)	URTD 3.7 (2.7–4.9)	URTD 3.5 (2.5–4.7)	Kidney failure 3.8 (2.1–6.6)
6	Lameness 2.9 (0.9–7.8)	Dermatitis or eczema 5.1 (4.6–5.6)	Lameness 3.2 (2.5–3.9)	Vomiting 3.9 (3.0–5.0)	Health services 3.3 (2.4–4.5)	Respiratory disorder 3 (2.1–4.2)	Lameness 3.5 (1.9–6.2)
7	Loss of appetite 2.9 (0.9–7.8)	Lameness 3.6 (3.1–3.9)	Wound 2.9 (2.3–3.6)	Gingivitis 3.1 (2.3–4.0)	Diarrhea 3.1 (2.2–4.2)	Health services 3 (2.0–4.1)	Diarrhea 3.2 (1.7–5.8)
8	Endoparasites 2.9 (0.9–7.8)	URTD 3.2 (2.8–3.6)	Skin Pruritus 2.9 (2.2–3.5)	Malassezia infection 2.8 (2.0–3.7)	Preventive medicine 2.8 (2.0–3.9)	Vomiting 2.8 (1.9–3.9)	Heart disease 2.9 (1.5–5.5)
9	Trauma 2.2 (0.5–6.8)	Trauma 3.1 (2.7–3.5)	Malassezia infection 2.7 (2.1–3.4)	Trauma 2.8 (2.0–3.6)	Malassezia infection 2.4 (1.6–3.4)	Preventive medicine 2.6 (1.7–3.7)	Preventive medicine 2.7 (1.3–5.1)
10	Lethargy 2.2 (0.5–6.8)	Malassezia infection 2.2 (1.9–2.6)	Trauma 2.5 (1.9–3.2)	Health services 2.3 (1.6–3.2)	Gingivitis 2.4 (1.6–3.4)	Diarrhea 2.4 (1.6–3.5)	Loss of appetite 2.7 (1.3–5.1)
11	Dermatitis or eczema 1.5 (0.2–5.7)	Skin Pruritus 2 (1.7–2.3)	Dermatomycoses 2.3 (1.8–3.0)	URTD 2.1 (1.4–2.9)	Heart disease 2.2 (1.4–3.2)	Kidney failure 2.4 (1.6–3.5)	Mammary tumor 2.7 (1.3–5.1)
12	Wound 1.5 (0.2–5.7)	Dermatomycoses 2 (1.6–2.3)	Neutering surgery 2.2 (1.6–2.8)	Skin Pruritus 2.1 (1.4–2.9)	Dermatomycoses 2.1 (1.4–3.1)	Pyoderma 2.1 (1.4–3.2)	Respiratory disorder 2.4 (1.1–4.7)
13	Parvovirus infection 1.5 (0.2–5.7)	Wound 1.8 (1.5–2.1)	Gingivitis 2 (1.5–2.6)	Pyoderma 1.9 (1.3–2.7)	Skin Pruritus 2 (1.3–3.0)	Disorders of orbit 2.1 (1.4–3.2)	Euthanasia 2.4 (1.1–4.7)
14	Coronavirus infection 1.5 (0.2–5.7)	Foreign body ingestion 1.8 (1.4–2.0)	Health services 1.7 (1.2–2.2)	Dermatomycoses 1.9 (1.2–2.6)	Neutering surgery 1.8 (1.1–2.7)	Cushing’s disease 2.1 (1.3–3.1)	Health services 2.1 (0.9–4.3)
15	Otitis externa 0.7 (0.04–4.6)	Patella luxation 1.4 (1.8–2.7)	Conjunctivitis 1.7 (1.2–2.2)	Conjunctivitis 1.9 (1.2–2.6)	Wound 1.8 (1.1–2.7)	Gingivitis 2 (1.2–3.0)	Cushing’s disease 2.1 (0.9–4.3)
16	Malassezia infection 0.7 (0.04–4.6)	Health services 1.4 (1.1–1.7)	Patella luxation 1.7 (1.2–2.2)	Patella luxation 1.7 (1.1–2.5)	Mammary tumor 1.8 (1.1–2.7)	Loss of appetite 2 (1.2–3.0)	Wound 1.8 (0.7–4.0)
17	Skin Pruritus 0.7 (0.04–4.6)	Conjunctivitis 1.4 (1.1–1.6)	URTD 1.5 (1.0–2.0)	Wound 1.6 (1.0–2.3)	Keratitis 1.6 (1.0–2.5)	Mammary tumor 2 (1.2–3.0)	Disorders of orbit 1.8 (0.7–4.0)
18	Patella luxation 0.7 (0.04–4.6)	Tooth extraction 1.1 (0.8–1.3)	Foreign body ingestion 1.5 (1.0–2.0)	Neutering surgery 1.5 (0.9–2.2)	Disorders of orbit 1.5 (0.8–2.3)	Malassezia infection 1.9 (1.2–2.9)	Trauma 1.5 (0.5–3.6)
19	Respiratory disorder 0.7 (0.04–4.6)	Pregnancy 1.1 (0.8–1.3)	Pregnancy 1.4 (1.0–1.9)	Foreign body ingestion 1.3 (0.8–1.9)	Respiratory disorder 1.4 (0.8–2.2)	Seizure 1.7 (1.0–2.6)	Skin Pruritus 1.5 (0.5–3.6)
20	Pyoderma 0.7 (0.04–4.6)	Ear pruritus 1 (0.8–1.2)	Ear pruritus 1.3 (0.9–1.8)	Mass/nodule 1.2 (0.8–1.9)	Pyoderma 1.4 (0.8–2.2)	Skin Pruritus 1.5 (0.9–2.4)	Dermatomycoses 1.5 (0.5–3.6)
21	Others 12.5 (7.6–19.5)	Others 26.7 (25.7–27.7)	Others 34.3 (32.5–36.1)	Others 37.5 (35.1–39.4)	Others 43.5 (40.6–46.2)	Others 42.7 (39.7–45.6)	Others 44.5 (39.2–50.0)

**Notes:**

Results are presented according to age group, during the one-year study period. URTD, upper respiratory tract disease.

### Major medical causes for visiting veterinary clinic by breeds

Table 2 shows the prevalence of common medical problems according to individual breeds. Briefly, for Malteses, the major reasons for visiting the clinics were preventive medicine 10.3% (95% CI [9.3–15.9]), otitis externa 8.8% (95% CI [7.9–9.7]), and dermatitis or eczema 6.9% (CI [6.1–7.7]). For Poodles, preventive medicine 14.5% (95% CI [13.0–15.9]) was most common reason for visiting vet clinic and the most common medical problem was otitis externa 8.3% (95% CI [7.2–9.4]), followed by diarrhea 6.5% (95% CI [5.5–7.5]) and vomiting 6.2% (95% CI [5.2–7.2]). Pomeranians had similar disorders which was compared with Poodles, while they showed a prevalence of upper respiratory tract disease 4.5% (95% CI [3.5–5.8]) that was higher than in other breeds. For Shih Tzus, the most cause for visiting vet clinic was dermatitis or eczema 9.3% (95% CI [7.7–11.1]), followed by otitis externa 8.2% (95% CI [6.7–9.9]) and preventive medicine 6.0% (95% CI [4.7–7.5]). In addition, diseases of the visual system, such as conjunctivitis, obit disorders, and keratitis, occurred more commonly in Shih Tzus than in other breeds. For mixed breeds, trauma (including injuries, poisoning, or other related prevalence than in other breeds. For Yorkshire Terriers, gingivitis 2.8% (95% CI [1.9–4.1]) and seizures 1.7% (95% CI [1.0–2.6]) were much more common than in other breeds. For all breeds, otitis externa, dermatitis or eczema, vomiting, diarrhea, lameness, Malassezia infection, and dermatomycoses were common medical problems.

**Table 2:** The top 20 most common reasons for canine patient visits to primary-care veterinary clinics.

	Percentage of each medical condition in the seven most common breeds (95% confidence interval)						
Rank	Maltese	Poodle	Pomeranian	Shih Tzu	Mixed	Yorkshire Terrier	Chihuahua
1	Preventive medicine 10.3 (9.3–11.3)	Preventive medicine 14.5 (13.0–15.9)	Preventive medicine 14.7 (12.8–16.6)	Dermatitis or eczema 9.3 (7.7–11.1)	Preventive medicine 13.4 (11.4–15.5)	Dermatitis or eczema 7.5 (5.9–9.2)	Preventive medicine 14.1 (11.4–17.0)
2	Otitis externa 8.8 (7.9–9.7)	Otitis externa 8.3 (7.2–9.4)	Diarrhea 5.6 (4.4–7.0)	Otitis externa 8.2 (6.7–9.9)	Dermatitis or eczema 5.8 (4.5–7.4)	Preventive medicine 6.9 (5.4–8.6)	Neutering surgery 5.4 (3.8–7.5)
3	Dermatitis or eczema 6.9 (6.1–7.7)	Diarrhea 6.5 (5.5–7.5)	Neutering surgery 5.4 (4.2–6.7)	Preventive medicine 6.0 (4.7–7.5)	Neutering surgery 5.3 (4.0–6.8)	Vomiting 5.8 (4.4–7.4)	Diarrhea 5.3 (3.7–7.4)
4	Vomiting 4.5 (3.9–5.2)	Vomiting 6.2 (5.2–7.2)	Vomiting 5.3 (4.1–6.6)	Diarrhea 4.1 (3.0–5.4)	Diarrhea 4.9 (3.7–6.4)	Otitis externa 5.7 (4.3–7.3)	Vomiting 4.2 (2.7–6.1)
5	Diarrhea 4.4 (3.8–5.1)	Neutering surgery 5.9 (4.9–6.9)	Lameness 4.6 (3.5–5.8)	Vomiting 3.9 (2.8–5.2)	Vomiting 4.9 (3.7–6.4)	Diarrhea 4.3 (3.1–5.8)	Lameness 3.7 (2.3–5.5)
6	Lameness 3.5 (2.9–4.1)	Dermatitis or eczema 4.9 (4.0–5.8)	URTD 4.5 (3.5–5.8)	Lameness 3.0 (2.1–4.2)	Trauma 4.0 (2.9–5.4)	Skin Pruritus 3.4 (2.4–4.7)	Otitis externa 3.5 (2.2–5.3)
7	Malassezia infection 3.4 (2.8–3.9)	Lameness 4.1 (3.3–4.9)	Dermatitis or eczema 4.2 (3.1–5.3)	Skin Pruritus 3.0 (2.1–4.2)	Lameness 3.8 (2.7–5.0)	Lameness 3.2 (2.2–4.5)	Dermatitis or eczema 3.4 (2.1–5.1)
8	Neutering surgery 3.3 (2.7–3.9)	Trauma 3.1 (2.4–3.8)	Trauma 4.1 (3.1–5.3)	Malassezia infection 2.6 (1.7–3.7)	Health services 3.0 (2.1–4.2)	URTD 3.2 (2.2–4.5)	Trauma 3.4 (2.1–5.1)
9	URTD 2.9 (2.3–3.4)	Malassezia infection 3.0 (2.3–3.7)	Otitis externa 3.4 (2.5–4.4)	Conjunctivitis 2.5 (1.7–3.6)	Otitis externa 2.9 (2.0–4.0)	Gingivitis 2.8 (1.9–4.1)	URTD 3.0 (1.8–4.7)
10	Dermatomycoses 2.3 (1.8–2.8)	URTD 2.4 (1.8–3.0)	Patella luxation 3.0 (2.1–4.0)	Neutering surgery 2.4 (1.6–3.5)	Skin Pruritus 2.8 (1.9–3.9)	Neutering surgery 2.6 (1.7–3.7)	Patella luxation 2.9 (1.7–4.5)
11	Health services 2.3 (1.8–2.3)	Wound 2.1 (1.6–2.8)	Health services 1.9 (1.2–2.8)	Disorders of orbit 2.4 (1.6–3.5)	Wound 2.2 (1.4–3.2)	Trauma 2.4 (1.5–3.5)	Foreign body ingestion 2.7 (1.6–4.4)
12	Trauma 1.9 (1.5–2.4)	Skin Pruritus 1.9 (1.3–2.5)	Respiratory disorder 1.7 (1.0–2.5)	URTD 2.3 (1.5–3.4)	Malassezia infection 2 (1.2–3.0)	Malassezia infection 2.2 (1.3–3.3)	Skin Pruritus 2.2 (1.2–3.8)
13	Patella luxation 1.9 (1.5–2.4)	Dermatomycoses 1.8 (1.3–2.4)	Pregnancy 1.5 (0.6–1.8)	Health services 1.8 (1.1–2.8)	URTD 1.7 (1.0–2.6)	Health services 2.1 (1.3–3.1)	Malassezia infection 1.8 (0.9–3.2)
14	Skin Pruritus 1.9 (1.5–2.3)	Foreign body ingestion 1.6 (1.1–2.2)	Malassezia infection 1.4 (0.5–1.7)	Keratitis 1.8 (1.1–2.8)	Conjunctivitis 1.6 (0.9–2.5)	Seizure 2.1 (1.3–3.1)	Health services 1.6 (0.8–3.0)
15	Conjunctivitis 1.7 (1.3–2.1)	Health services 1.6 (1.1–2.1)	Tooth extraction 1.4 (0.8–2.2)	Mass/nodule 1.7 (1.0–2.7)	Pyoderma 1.4 (0.8–2.3)	Wound 2.0 (1.2–3.0)	Disorders of orbit 1.6 (0.8–3.0)
16	Foreign body ingestion 1.7 (1.3–2.1)	ear pruritus, unspecified 1.4 (0.9–1.9)	Dermatomycoses 1.3 (0.8–2.1)	Foreign body ingestion 1.6 (0.9–2.5)	Gingivitis 1.3 (0.7–2.2)	Patella luxation 1.7 (1.0–2.7)	Nasal discharge 1.6 (0.8–3.0)
17	Wound 1.7 (1.2–2.1)	Patella luxation 1.3 (0.8–1.8)	Foreign body ingestion 1.3 (0.8–2.1)	Trauma 1.5 (0.8–2.3)	Loss of appetite 1.3 (0.7–2.2)	Dermatomycoses 1.6 (0.9–2.6)	Dermatomycoses 1.4 (0.7–2.8)
18	Heart disease 1.5 (1.1–1.9)	Gingivitis 1.2 (0.8–1.7)	Toxicity 1.2 (0.7–2.0)	Dermatomycoses 1.3 (0.7–2.1)	Alopecia 1.3 (0.7–2.2)	Disorders of orbit 1.5 (0.8–2.4)	Conjunctivitis 1.3 (0.6–2.6)
19	Gingivitis 1.5 (1.1–1.9)	Loss of appetite 1 (0.6–1.5)	Gingivitis 1.2 (0.6–1.9)	Gingivitis 1.2 (0.6–2.0)	Dermatomycoses 1.2 (0.6–2.0)	Conjunctivitis 1.4 (0.8–2.4)	Heart disease 1.3 (0.6–2.6)
20	Pyoderma 1.3 (0.9–1.7)	Toxicity 1.0 (0.6–1.4)	Skin Pruritus 1.1 (0.6–1.8)	Pyoderma 1.2 (0.6–2.0)	Respiratory disorder 1.2 (0.6–2.0)	Pyoderma 1.4 (0.8–2.4)	Keratitis 1.3 (0.6–2.6)
21	Others 32.3 (30.8–33.7)	Others 26.5 (2.4–2.8)	Others 31.4 (28.9–33.9)	Others 38.1 (35.3–41.0)	Others 34 (31.2–36.8)	Others 36.5 (33.5–39.5)	Others 34.5 (30.8–38.9)

**Notes:**

Results are shown according to breed group, during the one-year study period. URTD, upper respiratory tract disease.

### Statistical analysis

Table 3 shows the *p* values derived from Pearson’s chi-squared test for signs or diagnoses, according to the age groups and individual breeds. For the age groups, the majority of the top 20 presenting signs or diagnoses showed statistically significant differences among the groups, except for conjunctivitis, dermatomycoses, skin pruritus, foreign body ingestion, lameness, and Malassezia infection. For breeds, the majority of the top 20 presenting signs or diagnoses also showed statistically significant differences among the breeds, except for dermatomycoses, ear pruritus, health services, loss of appetite, and pyoderma.

**Table 3:** Statistically significant differences in presenting signs and diagnoses, according to age groups and breeds.

	Presenting signs and diagnoses	*p*-value for age	Presenting signs and diagnoses	*p*-value for breed
1	Conjunctivitis	0.406	Alopecia	**0.000**
2	Coronavirus infection	**0.000**	Conjunctivitis	**0.000**
3	Dermatitis or eczema	**0.000**	Dermatitis or eczema	**0.000**
4	Dermatomycoses	0.056	Dermatomycoses	0.500
5	Diarrhea	**0.000**	Diarrhea	**0.006**
6	Disorders of orbit, unspecified	**0.001**	Disorders of orbit, unspecified	**0.000**
7	Ear pruritus, unspecified	**0.000**	Dyspnea	**0.013**
8	Endoparasite	**0.000**	Ear pruritus, unspecified	0.330
9	Skin Pruritus	0.088	Skin Pruritus	**0.001**
10	Foreign body ingestion	0.494	Foreign body ingestion	**0.000**
11	Gingivitis	**0.000**	Gingivitis	**0.000**
12	Health services	**0.000**	Health services	0.124
13	Heart disease	**0.000**	Heart disease	**0.000**
14	Cushing’s disease	**0.000**	Keratitis	**0.000**
15	Keratitis	**0.000**	Lameness	0.362
16	Kidney failure	**0.000**	Loss of appetite	0.144
17	Lameness	0.355	Malassezia infection	**0.001**
18	Lethargy	**0.000**	Mammary tumor	**0.000**
19	Loss of appetite	**0.000**	Mass	**0.029**
20	Malassezia infection	0.235	Nasal discharge	**0.001**
21	Mammary tumor	**0.000**	Neutering surgery	**0.000**
22	Mass	**0.000**	Otitis externa	**0.000**
23	Neutering surgery	**0.000**	Patella luxation	**0.000**
24	Otitis externa	**0.000**	Pregnancy	**0.021**
25	Parvovirus infection	**0.000**	Preventive medicine	**0.000**
26	Patella luxation	**0.000**	Pyoderma	0.246
27	Pregnancy	**0.000**	Seizure	**0.000**
28	Preventive medicine	**0.000**	Tooth extraction	**0.024**
29	Pyoderma	**0.000**	Toxicity	**0.000**
30	Seizure	**0.000**	Trauma	**0.000**
31	Tooth extraction	**0.000**	Upper respiratory tract disease	**0.001**
32	Trauma	**0.001**	Vomiting	**0.028**
33	Upper respiratory tract disease	**0.000**	Wound	**0.000**
34	Vomiting	**0.000**		
35	Wound	**0.000**		

**Note:**

The most common reasons presented in Tables 1 and 2 are included. *p*-value <0.05 (bold font) is considered statistically significant.

## Discussion

### Data sources

There are several previous reports that have used quantitative surveys to investigate morbidity or mortality risk factors for diseases (Bonnett & Egenvall, 2010; Bonnett et al., 2005; Dorn, 2000; Egenvall et al., 2005; O’Neill et al., 2014). For these types of surveys, a variety of data sources can be used to examine disease prevalence patterns. Veterinary teaching hospitals, referral practices with specialists, primary-care veterinary clinics, insured animal data, Kennel club registrations, questionnaires, and internet-based surveys are commonly used to conduct demographic or health condition research in companion animals (Bonnett & Egenvall, 2010; Diez et al., 2015; Dorn, 2000; Egenvall et al., 2005; Gobar, Case & Kass, 1998; Kubinyi, Turcsán & Miklósi, 2009; O’Neill et al., 2014; Zur, Lifshitz & Bdolah-Abram, 2011). Veterinary EMR data have been proposed as a statistically valuable source of information for clinical research in dogs (O’Neill et al., 2014, 2015; Sánchez-Vizcaíno et al., 2015). EMR data have a number of benefits: they are not difficult to acquire, they provide a wide range of comprehensive information, and they allow a wide range of multi-dimensional analyses. Currently, most veterinary clinics in ROK use EMRs for their practice management. The system is convenient for storing and sorting patient information, and it is easy to search desired categorical information. Notably, there were >400 signs, diagnostic terms, and descriptions of owners’ concerns in the collected data. For these reasons, some of the standard terms could be not used in the traditional manner. For example, “heart disease” was used when recorded terms were cardiomegaly, heart failure, valve disease, and cardiac murmurs, while upper “respiratory tract disease” included presenting signs or diagnoses such as cough and sneezing.

The International Classification of Diseases is the standard diagnostic tool for epidemiology, health management and clinical purposes; it is used to monitor the incidence and prevalence of diseases and other health problems, providing a general representation of population-level health status in human medicine. In veterinary medicine, several previous attempts have been undertaken with regard to standardization of diagnostic tools for canine diseases, as well as the presentation of clinical signs or/and diagnostic terms for epidemiology (Inoue et al., 2015; Martini et al., 2016; Merlo et al., 2008). Hence, we used the ICD category system from WHO for disease classification to reduce confusion associated with the meanings of the terms. Technically, we used the ICD categories to analyze the collected data at the top level, according to organ systems (chapters). The most common diseases, according to ICD classification, were “Diseases of the skin and subcutaneous tissue.” This result was same at the level of presenting signs or diagnoses, where dermatitis or eczema was the most common medical problem for both age and breeds in this study.

### Age distribution

In this study, there was a lower proportion of <1-year-old dogs (0.9%) then we expected. The most common age for visiting vet clinics was 1–3 years (53.0%); dogs aged ≥10 years old (17.3%) also showed a high proportion. In a previous study, most dogs that visited vets were the <1-year-old or 4-to-7-year-old groups in the USA (Lund et al., 1999); a demographic study in the UK showed that 0-to-1-year-old dogs were the highest proportion, with clinic visits slightly decreasing with increasing age (Sánchez-Vizcaíno et al., 2017). These results are different than our study in that the <1-year-old population was a lower proportion of our total. The average age was 4.8 years and the oldest age was 20 years in this study. Most researchers have insisted that the average age of pet dogs has been increasing, and that their expected lifespan is longer (Egenvall et al., 2005; O’Neill et al., 2013). This study shows that the proportion of >10-years-old dogs was higher than the proportion of <1-year-old dogs. These older dogs will be an important part of veterinary medicine; with their different prevalences of diseases compared with younger dogs, owners and veterinarians will need health care plans that take properly consider the age status of the dogs.

### Breed distribution

The most popular breed was Maltese, followed by Poodle, Pomeranian, Shih Tzu, Yorkshire Terrier, Chihuahua, and mixed breed. These seven breeds comprised 74.7% of the total dogs in this study. Approximately 24.1% of the population own dogs, and approximately 6.62 million dogs are present in the ROK (Animal and Plant Quarantine Agency, 2017). In a recent survey (Proportionate quota sampling, Online survey) of major cities in the ROK, including Seoul and six other metropolitan cities, Maltese was the most popular breed (21.2%), followed by Shih Tzu (11.7%), poodle (11.4%), mixed breed (6.1%), Yorkshire terrier (5.2%), and Pomeranian (5.1%) (Korean Pet Food Association, 2017). Notably, small dogs are the major varieties, consistent with the results of the present study. However, due to the limited methodology of the data collection, it might not reflect all small breed dogs in the ROK. Several studies found a general trend for small breeds to have a longer expected life span than large breeds (Egenvall et al., 2005; Fleming, Creevy & Promislow, 2011; O’Neill et al., 2013; Proschowsky, Rugbjerg & Ersbøll, 2003). In addition, disease prevalences were affected by breed (Dorn, 2000; Harvey, 1998; Lund et al., 1999; Miller et al., 2016). Because most breeds in ROK are small and toy breeds, veterinarian practices will need to focus on the conditions that most affect these breeds. Additionally, knowledge of the risk of various diseases in these breeds is very useful in developing a differential diagnosis and selecting treatments more effectively.

### The major medical causes for visiting veterinary clinic

This study revealed the major medical disorders in dogs visiting primary-care veterinary clinics in ROK. The most cause for visiting veterinary clinic was for preventive medicine and the most commonly presented sign was dermatitis or eczema, followed by otitis externa, diarrhea, and vomiting. Previous reports have shown the most common reasons for visiting vet clinics were: skin lump, pruritus, vomiting, loss of appetite, lameness, and diarrhea (Robinson et al., 2015); otitis externa, periodontal disease, and anal sac impaction (O’Neill et al., 2015); and dental calculus, gingivitis, and otitis externa (Lund et al., 1999). Compared with previous study, the causes of visiting veterinary clinics were similar, but there were some differences in their rank levels in their calculations. Notably, dental diseases, such as gingivitis, were significantly lower in our study.

The most affected organ systems were the integument, digestive, and musculoskeletal systems in the study by O’Neill et al. in the UK (2015), whereas digestive disease was most common, followed by dermatological disorders and cardiovascular disease in a study in Italy (Martini et al., 2016). In our study, skin diseases were most common, followed by digestive diseases, preventive medicine, ear diseases, and diseases of the visual and musculoskeletal systems. Even though the ratios were different in each study, the main causes (skin, digestive, and ear problems) were similar; although in the Italian study, cardiovascular diseases were much lower than in our study.

### Major medical causes for visiting veterinary clinic by age groups

Previous reports suggest that the prevalence of disease is affected by age, as well as by breed (Bonnett & Egenvall, 2010; Egenvall et al., 2005). We analyzed the frequency and distribution of medical problems by life stage (<1 year of age and every three years, from one to 16 years). Young dogs, for example, are more easily exposed to infectious diseases, such as parvovirus or coronavirus, and intestinal parasites, even though the proportion of preventive medicine is higher, and the prevalence of trauma such as falls, accidents, and factures is also higher than in older age groups. The prevalence of progressive diseases, such as neoplasm, heart diseases, and arthritis is related to age group as well (Bonnett & Egenvall, 2010; Egenvall et al., 2005). This study shows that some kinds of progressive or degenerative diseases had a higher prevalence in older groups than in younger groups, including circulation disorders (e.g., heart disease), neoplasms (e.g., mammary tumors), musculoskeletal disorders (e.g., lameness), and hormonal disorders (e.g., Cushing’s disease). The prevalence of heart diseases increases with age in small breed dogs, and dogs older than eight years have a reduced survival time, compared to younger dogs (Guglielmini, 2003; Templeton et al., 1976). A previous study showed the mean age for dogs with heart disease was 10.6 ± 2.62 years. This study revealed a similar result, in which the prevalence of heart disease was significantly different, according to age (*p* < 0.05). Additionally, heart disease is a high-risk factor in terms of mortality in dogs (Bonnett & Egenvall, 2010; Egenvall et al., 2005; Hoummady et al., 2016). Age-related changes of cardiovascular function in dog affect the prevalences of not only hypotension, blood velocity, arterial compliance, and dispensability (Haidet et al., 1996; Miller, Nealeigh & Crowder, 1976), but also increased ventricular systolic and diastolic stiffness associated with prolonged duration of myocardial contractility (Templeton et al., 1976). In dogs, heart disease has been related to serious conditions, including progressive heart failure, pulmonary edema and/or congestion, hypertension, left arterial rupture, and pericardial tamponade (Borgarelli et al., 2008; Guglielmini, 2003; Pouchelon et al., 2015). These reasons might reflect the result that increasing the prevalence of respiratory disorder in presented study. Some types of canine patients with class I heart disease have no clinical signs that are evident, even during exercise. However, this disease status can change frequently and dramatically over short periods of time (Guglielmini, 2003). In older dogs, especially those >10 years, regular heart exams that may include heart rate, auscultation, radiography, and echocardiogram, are recommended.

Previous studies have described neoplasia as the most important risk factor for mortality in older dogs at 16.6% (O’Neill et al., 2013), 27.0% (Adam et al., 2010), 14.5% (Proschowsky, Rugbjerg & Ersbøll, 2003), and 17.8% (Bonnett et al., 2005). In addition, tumors of the mammary gland were the most common type, with a mean percentage of malignancy of 50.9% in female dogs (Kustritz, 2007; Sorenmo, 2003). Increasing age is the most significant risk factor for the development of mammary gland tumors in dogs, with a mean age at diagnosis of approximately 8.4–10 years (Chang et al., 2005; Kustritz, 2007). We also observed increased rates of mammary tumors in the >10 years old age groups. Sexually intact status is a major risk factor for the development of mammary gland tumors; sexually intact dogs have a seven-fold increased risk for developing mammary gland tumors with increasing age, compared with the risk for spayed dogs (Misdorp, 1988; Kustritz, 2007). Hence, as age increases, unneutered female dogs should have regular health checkups, especially with regard to mammary gland tumors.

One of most interesting findings of this study was that as the age increased, the proportion of preventive medicine became relatively low (*p* < 0.05). This suggests that prevention of infectious disease occurs predominantly at younger ages and tends to be neglected as dogs grow older. In one report, a significant proportion of dogs had no vaccinations (32%) and no prevention against external parasites (47%) or internal parasites (31%) during a campaign for improving preventive medicine in Belgium, even though these are important preventive measures that affect public health and quality of life in pets (Diez et al., 2015; Schultz et al., 2010). Regular preventive medicines would help decrease mortality and morbidity, especially for some kinds of infectious disease, including parasitic diseases like heartworm and tick-borne diseases; it has benefit for prevention of human-animal interacted diseases.

Diseases of the digestive system are the most common diseases across systemic categories and are responsible for a high proportion (14.5%) of causes of death, especially in dogs under 3 years of age (O’Neill et al., 2013). Diarrhea and/or vomiting were the most common signs in gastrointestinal diseases, which include both infectious and non-infectious etiologies (Sánchez-Vizcaíno et al., 2015). In our results, vomiting (5.0%) and diarrhea (5.2%) accounted for a high proportion of all medical problems and the total proportion of diseases of the digestive system was 14.0%. Presenting signs of these diseases in dogs <1 year of age (diarrhea 11.8%, vomiting 5.1%) were higher than in older groups, but as a category, “Diseases of the digestive system” was ranked higher than other systemic disease categories in all age groups. Gastrointestinal disease was the most important factor in the prevalence of disease in young and adult dogs overall (Fleming, Creevy & Promislow, 2011).

Dermatitis and otitis externa have been reported as the most common health problems in dogs (O’Neill et al., 2014; Lund et al., 1999; Martini et al., 2016). These diseases can begin as early as 1 year of age and are considered major diseases; they occurred at a high rate across both breeds and age groups in this study. The most common presenting signs or diagnoses of skin disease were pruritus, swellings, and alopecia in a previous study (Hill et al., 2006). In this study, dermatitis or eczema (6.4%) was the most common sign followed by erythema and/or pruritus, dermatomycoses, pyoderma, mass and/or nodule, alopecia, and allergic dermatitis. Notably, the signs of atopic dermatitis were exhibited by 95% of the dogs less than five years of age (Zur et al., 2002). Additionally, 55–60% of dogs diagnosed with atopic dermatitis have otitis externa (Zur et al., 2002; Zur, Lifshitz & Bdolah-Abram, 2011). Based on these results, if a dog has presenting signs or diagnoses related to the skin, it is necessary to check for ear diseases.

Dental diseases occur more commonly in small breed dogs than in large breeds, and the rate increases with age (Kyllar & Witter, 2005). In a previous study, periodontal diseases and calculus were the most common oral diseases in the dog (Page & Schroeder, 1981). These diseases are considered to be associated with diet, breed, and aging (Harvey, 1998; Lund et al., 1999). Although tooth brushing, dental scaling and polishing removes plaque and calculus from all tooth surfaces, one study confirmed that as soon as tooth brushing stops, gingival inflammation develops rapidly (Ingham & Gorrel, 2001). Continuous periodontal care is an important factor for managing oral problems. Small animal practitioners should perform more thorough checkups of the oral cavity because our study showed a low rate of dental diseases than expected, even though it is known that the prevalence of dental disease is quite high (Lund et al., 1999; O’Neill et al., 2014).

### Major medical causes for visiting veterinary clinic by breeds

We analyzed the frequency and distribution of medical problems in six breeds (Maltese, Poodle, Pomeranian, Shih Tzu, Yorkshire Terrier, and Chihuahua) and in mixed breed dogs. Although previous reports have demonstrated various breed distributions of medical conditions, our results clearly differ from those regarding distributions of dog breeds in other countries. The main breeds in ROK are small dogs and toy dogs. Common causes for visiting the veterinary clinic were similar across these breeds: preventive medicine, otitis externa, dermatitis or eczema, and diarrhea and vomiting. These medical problems, manifested as brown ears (externa otitis), itching on skin, or diarrhea and vomiting, are simple for owners to recognize, which is reflected in our results. However, some of these health problems are not obvious to the owner and can only be detected with by veterinary diagnosis. Notably, purebred dogs of many breeds and some mixed breed dogs are prone to congenital or heritable diseases (Asher et al., 2009; Dorn, 2000). In pedigree dogs, hip dysplasia, patella luxation, entropion, retinal atrophy, elongated soft palate, skin fold dermatitis, uterine inertia, elbow dysplasia, lens luxation, ectropion, and trichiasis have been commonly identified (Asher et al., 2009; Hodgman, 1963). As dogs aged, skin diseases, including dermatitis or eczema, malassezia infection in skin, dermatomycoses, skin pruritus, and pyoderma show high prevalence across breeds. We did not know which problems were heritable diseases; however, a previous report noted that some purebred dogs could be affected by various endogenous risk factors, such as hyperadrenocorticism in Poodle, hypothyroidism in Doberman Pinscher, and zinc-responsive skin diseases in Cocker Spaniel and Alaskan malamute (Miller et al., 2013; Scott & Paradis, 1990).

These reports showed a genetic predisposition for the development of skin disease; however, skin diseases, especially allergic or atopic dermatitis, are suspected to be caused by environmental, rather than genetic, factors (Hillier & Craig, 2001). A previous study found that the major common causes of atopic dermatitis were house dust mites, molds, house dust, trees, weeds, and insects (Zur et al., 2002). Notably, it is difficult to detect early skin disease in long hair breeds, such as the major breeds of this study (Maltese, Toy Poodle, Pomeranian, Shih Tzu, and Yorkshire Terrier). The estimated proportion of dogs with allergic or atopic dermatitis might be under-estimated by primary veterinary practices because mild cases are often well-managed with symptomatic therapy without a specific diagnosis. Additionally, the prevalence of canine atopic dermatitis could be variably affected by type of veterinary practice, geographical region, survey methodology, study population selection, and/or criteria for establishing a diagnosis of disease (Hillier & Craig, 2001).

Otitis externa was more frequently observed in Maltese, Poodle, Shih Tzu, and Yorkshire Terrier breeds, which may be related to respective breed characteristics. Otitis externa is known to be related to skin diseases; it exhibits breed predisposition, with excessive hair in and around ear, typically in long-coated breeds (Hayes, Pickle & Wilson, 1987; Strain, 1996). The incidence of otitis externa in this study supports the assumption that it is a breed-related condition (*p* < 0.001).

We observed that, for Shih Tzu, orbital problems, including conjunctivitis, disorder of the orbit (unspecified), and keratitis were more frequent than in other breeds; we suspect that this is because of persistent irritation of the cornea or conjunctiva by eyelashes or facial hair, similar to congenital malformations of hair (e.g., trichiasis, median canthus, or aberrant cilia). Additionally, conformation character, such as median entropion, tends to be more common in brachycephalic breeds with prominent nasal folds, including Shih Tzu, Pekingese, English Bulldog, and Pug (Christmas, 1992).

Gonadectomy of small and mixed breed dogs were observed at high proportions in the top 10 breeds. These surgeries are commonly recommended as prevention for genital disorders, such as pyometra, mammary tumors, testicular tumors, and prostatic neoplasia and behavioral problems, such as mounting and urine spraying (Kustritz, 2007). However, in house dogs, overweight or obese statuses were highly prevalent among neutered dogs; the most commonly reported risk factor for obesity was gonadectomy (Asher et al., 2009). Even though we did not analyze body weight, it is an important factor contributing to life quality and longevity in companion dogs (Fleming, Creevy & Promislow, 2011; O’Neill et al., 2013). A limitation of this study is that it is not a comprehensive, representative survey of the entire ROK. It is considered to be relevant for indoor dogs living in big cities, because it is aimed at primary veterinary clinics in Seoul, the largest city in ROK, and Jeonju, a smaller city. Therefore, it is not likely to be applicable to rural areas. Furthermore, if signs alone were considered when observing or consulting cases in veterinary clinics, some of the most frequently encountered breed-specific and congenital problems, and a variety of diseases that result from conformation character, malformation, and/or endocrine disorders would be missed because owners might not recognize them or regard them as important. Understanding why these types of problems tend to occur at higher rates than in other breeds may help in deciding when they should be prioritized; for owners, regular visits to the veterinarian for health screening can help identify these undetected problems and allow better treatment and management of some problems in earlier stages.

## Conclusions

The results of this study provide various information regarding the cause of visiting veterinary clinic for dogs by age and breeds. Because most of the dog breeds in ROK are small and toy breeds, which are expected to have a long lifespan, we need to design appropriate approaches for them. The information in this study could provide information to educate owners, improve strategies for disease prevention, and enable management of problems earlier and more effectively. Ideally, it would be helpful to establish guidelines for primary health care at the national level. Additionally, various dimensions of analysis are required to determine the trends for factors affecting disease patterns. To our knowledge, no other study has performed this type of analysis using comparable methods to collect EMR data from a large sample of dogs in the ROK. This is the first study that describes disease patterns and occurrences according to age. There is a need for further studies to provide a more comprehensive analysis according to age groups within breeds.

## Supplemental Information

10.7717/peerj.5161/supp-1 Supplemental Information 1 Raw data for canine patient visits to primary-care veterinary clinics. We mixed all collected data from eleven veterinary clinics; repeated ongoing events within individual cases were excluded. A single line in the file holds the categories (heads) describing each type of data information in the raw data file.
Data: Dates of visits to the veterinary clinic; Case Number (given by author); Breed, Age, Sex.
Presenting signs and diagnoses: terms provided by the authors during analysis; if a patient had more than one disease, we separated them on individual lines.
ICD category: categories provided by the authors during analysis; if a patient had more than one disease, we separated them on individual lines.

10.7717/peerj.5161/supp-2 Supplemental Information 2 Supplemental file for Figs. 1–4. First sheet includes information about age profile for Fig. 1; Second sheet includes information about breed profile for Fig. 2; Third sheet includes information about ICD category for Fig. 3; Fourth sheet includes information about presenting signs or diagnoses for Fig. 4.

## Additional Information and Declarations

### Data Availability

The following information was supplied regarding data availability: The raw data are provided in the Supplemental Files.
